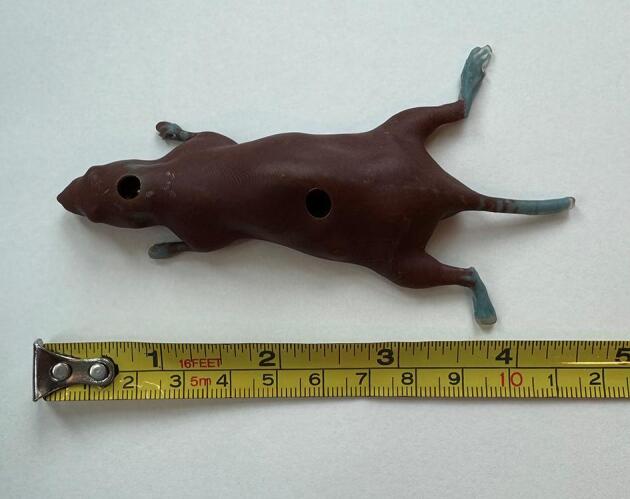# A comparative analysis of preclinical computed tomography radiomics using cone-beam and micro-computed tomography scanners

**DOI:** 10.1016/j.phro.2024.100615

**Published:** 2024-07-23

**Authors:** Kathryn H. Brown, Brianna N. Kerr, Mihaela Pettigrew, Kate Connor, Ian S. Miller, Liam Shiels, Colum Connolly, Conor McGarry, Annette T. Byrne, Karl T. Butterworth

**Affiliations:** a Patrick G. Johnston Centre for Cancer Research, Queen’s University Belfast, Belfast, United Kingdom; b Department of Physiology and Medical Physics and Centre for Systems Medicine, Royal College of Surgeons in Ireland, Dublin 2, Ireland; c National Preclinical Imaging Centre, Royal College of Surgeons in Ireland, Dublin 2, Ireland; d Northern Ireland Cancer Centre, Belfast Health & Social Care Trust, Belfast, United Kingdom

**Keywords:** Radiomics, Preclinical, Computed tomography, Cone-beam, Micro, Phantom, Tissue density, Reliability

## Abstract

**Background and purpose:**

Radiomics analysis extracts quantitative data (features) from medical images. These features could potentially reflect biological characteristics and act as imaging biomarkers within precision medicine. However, there is a lack of cross-comparison and validation of radiomics outputs which is paramount for clinical implementation. In this study, we compared radiomics outputs across two computed tomography (CT)-based preclinical scanners.

**Materials and methods:**

Cone beam CT (CBCT) and µCT scans were acquired using different preclinical CT imaging platforms. The reproducibility of radiomics features on each scanner was assessed using a phantom across imaging energies (40 & 60 kVp) and segmentation volumes (44–238 mm^3^). Retrospective mouse scans were used to compare feature reliability across varying tissue densities (lung, heart, bone), scanners and after voxel size harmonisation. Reliable features had an intraclass correlation coefficient (ICC) > 0.8.

**Results:**

First order and GLCM features were the most reliable on both scanners across different volumes. There was an inverse relationship between tissue density and feature reliability, with the highest number of features in lung (CBCT=580, µCT=734) and lowest in bone (CBCT=110, µCT=560). Comparable features for lung and heart tissues increased when voxel sizes were harmonised. We have identified tissue-specific preclinical radiomics signatures in mice for the lung (133), heart (35), and bone (15).

**Conclusions:**

Preclinical CBCT and µCT scans can be used for radiomics analysis to support the development of meaningful radiomics signatures. This study demonstrates the importance of standardisation and emphasises the need for multi-centre studies.

## 1 Introduction

Radiomics analysis translates medical images into quantitative data, termed “features”, in attempt to provide more information to improve patient diagnosis and treatment decision-making [1], [2]. Within oncology, radiomics has been proposed as a virtual biopsy to non-invasively determine the biological features of tumours including mutation status, immune response, and hypoxia [3]. Studies have shown that the implementation of radiomics features with clinical factors can improve patient risk stratification for overall survival and local regional control [4], [5].

Despite the advantages of radiomics image analysis, the question of feature reliability between scanners remains a major limitation in the transferability of radiomics outputs and their adoption into the clinic [6], [7]. To date, most radiomics studies are limited to a single-centre analysis and lack multi-centre validation, slowing its integration into clinical workflows [8], [9]. Multiple parameters feed into inter-scanner variability, including but not limited to, imaging modality, acquisition parameters, image discretization, reconstruction techniques and filtering [10], [11], [12].

In the past decade, preclinical imaging modalities have evolved which are downscaled in size and imaging energy [13]. These include preclinical computed tomography (CT), magnetic resonance (MR), and positron emission tomography (PET)-based scanners which have been routinely implemented for radiotherapy treatment planning, and detection of metastases or toxicities within translational research [14]. Recent studies have shown that these rich preclinical datasets could expand our current understanding of radiomics imaging signatures [15], [16], [17], [18], [19]. Like clinical studies, there is yet to be a comparison of preclinical radiomics outputs across imaging modalities and institutions.

Our study aimed to compare radiomics outputs from two preclinical CT-based scanners. This represents the first cross-centre comparison of radiomics outputs in preclinical research, shedding light on the transferability of radiomics features derived from both CBCT and µCT scanners.

## 2 Materials and methods

### 2.1 Imaging

#### 2.1.1 CBCT scans

Routine cone beam CT (CBCT) imaging was performed using the Small Animal Radiation Research Platform (SARRP, Xstrahl Life Sciences, Camberley, UK) at Queen’s University Belfast (Supplementary Table 1). The SARRP acquires images with tube voltages between 40 and 80 kVp. For this study, scans were acquired at energies routinely used for preclinical imaging (40 and 60 kVp) with a current of 0.8 mA, 60 s imaging time and 48 mAs current-exposure time [20]. Image reconstruction was performed using 360° images and filtered back-projection. Log(white/x) was applied to input images using FDK with a Hamming filtering window. CBCT scans had a slice thickness of 0.26 mm as the standard protocol for this scanner.

#### 2.1.2 Micro-CT scans

Routine µCT imaging was performed using the Quantum GX2 CT system (Perkin Elmer, UK) at the Royal College of Surgeons in Ireland (RCSI) (Supplementary Table 1). Scans were acquired using a standardised imaging protocol at energies of 40 and 60 kVp with a current of 0.088 mA, imaging time of 4 min and 21.12 mAs current-exposure time. Image reconstruction was performed using 360° images and filtered back-projection with post-filtering. µCT scans had a slice thickness of 0.09 mm as the standard protocol for this scanner.

### 2.2 Phantom

An anatomically correct mouse phantom developed by the National Physical Laboratory (NPL, UK) for preclinical dosimetry was used to assess intra-scanner reliability at different imaging energies and across segmentation volumes (Supplementary Fig. 1). This phantom has a unique design that is anatomically correct to a mouse with tissue-equivalent inserts with varying densities for bone (1.39 g/cm^3^), lung (0.68 g/cm^3^), and soft tissue (1.01 g/cm^3^) [21], [22]. The phantom was scanned twice on each scanner (scan-rescan) to assess the reproducibility of radiomics features between scans.

### 2.3 Mouse models

Retrospective mouse scans from both scanners were analysed and compared. All previous *in vivo* experimental procedures were carried out in accordance with the Home Office Guidance (Scientific Procedures Act 1986 (PPL2813)) and Directive 2010/63/EU of the European Parliament on protection of animals used for scientific purposes. For CBCT, 13 scans of female immunocompromised SCID mice aged 8–10 weeks (18 – 24 g) were used for comparative analysis. Mice were anaesthetised for imaging using with ketamine and xylazine (100 mg/kg and 10 mg/kg). For µCT, 13 scans of 7 males and 6 females of NOD SCID Gamma (NOD.Cg-Prkdc^scid^ Il2rgtm1Wj) mice aged 8-weeks old (20–25 g) were used for comparative analysis. Mice were anaesthetised using inhalant isoflurane for imaging.

### 2.4 Segmentation

All segmentations were created using ITK-SNAP software (version 3.8.0, http://www.itksnap.org) [23]. For phantom analysis, manual spherical contours were created using the 3-D round brush. To avoid variability, set brush sizes were used of 44, 92, and 238 mm^3^. For all mouse scans, tissues were contoured by two independent, experienced observers (KHB and BNK). Lung and bone (ribcage and spine) tissues were contoured using semi-automated methods as previously reported in Brown *et al* [24]. Mouse hearts were manually contoured using the brush tool. All segmentations were inspected and manually altered if required prior to analysis. These segmentations defined a volume of interest (VOI) for radiomics analysis.

### 2.5 Radiomics analysis

**Fig. 1:**
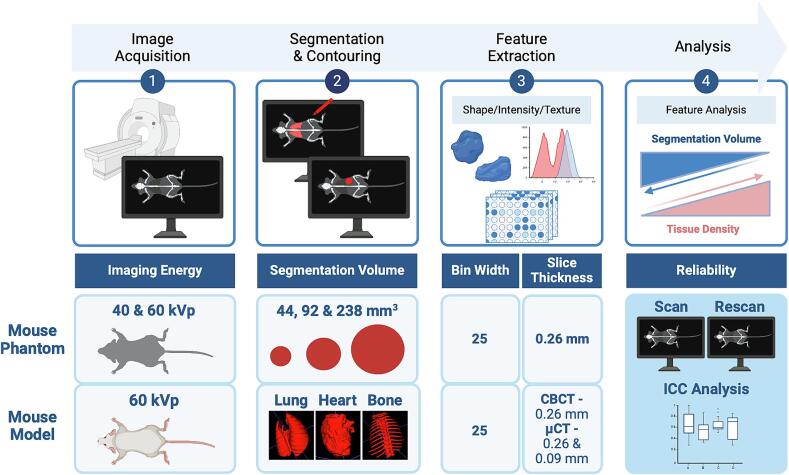
Overview of the radiomics workflow for comparison of preclinical CBCT and µCT scanners using various analytical parameters. An anatomically correct mouse phantom was used to assess the reproducibility of features between scans (scan-rescan) and also the impact of different segmentation volumes on feature reliability. Feature reliability in three tissues of different densities (lung, heart, and bone) was also assessed across two cohorts of mice scanned on either the CBCT or µCT scanner. Created with BioRender.com.

Radiomics features were extracted using PyRadiomics software (version 2.7.7, Harvard Medical School, USA), with a fixed bin width of 25 as previously reported [19]. A total of 842 features were extracted including original and wavelet feature types. All shape features were removed as these features could potentially confound the results from standard spherical segmentations or from tissue segmentations with distinct differences in the size, shape and anatomy. A total of 828 features were used for analysis including: first order statistics, gray level cooccurrence matrix (GLCM), gray level run length matrix (GLRLM), gray level size zone matrix (GLSZM), gray level dependence matrix (GLDM), and neighbouring gray tone difference matrix (NGTDM) [25]. Harmonisation of voxel sizes was compared by altering the resampledPixelSpacing of 0.09 mm to 0.26 mm for µCT (Fig. 1).

### 2.6 Statistical analysis

The intraclass correlation coefficient (ICC) score was used to determine the reliability of radiomics outputs. Reliability is defined as the extent outputs can be replicated [26]. ICC scores were calculated where an output of 0 indicated no reliability and 1 indicated perfect reliability [27]. ICC results are classified by Koo *et al* as poor (<0.5), moderate (0.5 – 0.7), good (0.7 – 0.9), and excellent (>0.9) [27], [28]. Reliable features were defined as those with an ICC score >0.8 to better match with previous thresholds reported in test–retest analysis. ICCs were calculated using the *irr* library from the *lpSolve* package in RStudio software (version 4.1.2) based on a single value with absolute-agreement and determined using 2-way mixed-effects models [27]. Scan-rescan analysis was completed on both scanners to assess reliability for each individual scanner at imaging energies of 40 and 60 kVp. Other parameters were also assessed for reliability including varying segmentation size, slice thickness and across mouse tissues [27]. Features that were identified as reliable for mouse tissues were then compared across scanners. Statistical analysis of the percentage of overlapping features was performed by two-way ANOVA using GraphPad Prism 7 (Version 7.01, San Diego, CA, USA) (www.graphpad.com), with significance reported as *p* ** <0.01.

## 3 Results

### 3.1 Assessment of scanner reproducibility using a phantom model

**Fig. 2:**
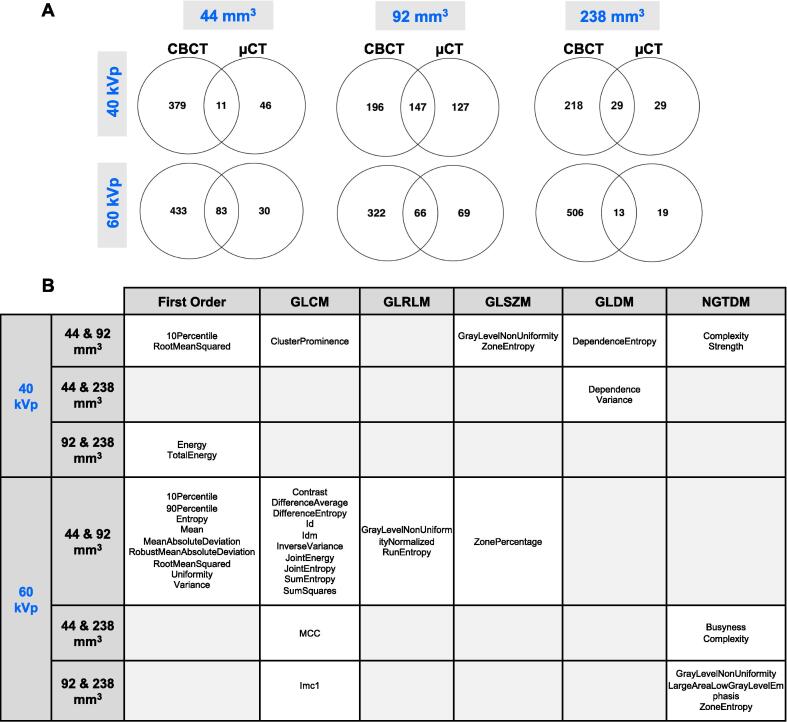
Comparative analysis of reliable *in phantom* radiomics features (ICC>0.8) using CBCT and µCT scanners. Scan-rescan tests were conducted on both scanners and the radiomics features were extracted from segmentation volumes of 44, 92, and 238 mm^3^ at 40 and 60 kVp. Venn diagrams in **Panel A** show the number of overlapping reliable features between CBCT and µCT scanners. **Panel B** summarises the overlapping feature classes across multiple segmentation volumes at 40 and 60 kVp.

The reliability of radiomics features on two CT-based preclinical scanners was determined through phantom scan-rescan analysis. Reliable features (ICC>0.8) from CBCT and µCT phantom scans were derived at two imaging energies (40 and 60 kVp) and three segmentation volumes (44, 92, and 238 mm^3^) (Fig. 2, Supplementary File 1). Overall, more radiomics features were reliable from the CBCT scans in comparison to µCT. A higher imaging energy increased the number of reliable features for CBCT but not for µCT. Increasing the segmentation volume did not increase the number of overlapping reliable radiomics features between scanners. First order and GLCM features are the most stable feature types across scanners and segmentation volumes (Fig. 2B).

### 3.2 The impact of tissue density on the reliability of radiomics features

**Fig. 3:**
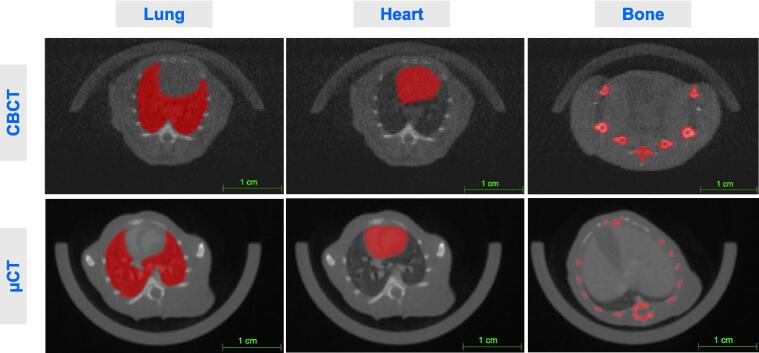
Representative scans for both CBCT and µCT scanners. Axial imaging panels with segmentations for lung, heart and bone are shown in red. (For interpretation of the references to colour in this figure legend, the reader is referred to the web version of this article.)

**Fig. 4:**
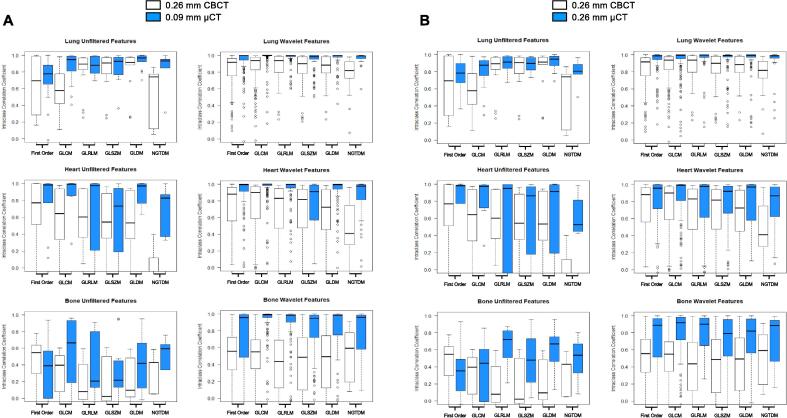
Boxplots comparing the ICC scores for all radiomics features derived from CBCT and µCT at slice thicknesses at image acquisition of 0.26 mm and 0.09 mm **(Panel A)** or at resampled voxel size of 0.26 mm **(Panel B)**. Features are categorised by feature class and feature type, unfiltered (left) and wavelet (right), for lung, heart, and bone.

The reliability of radiomics features at different tissue densities was assessed using retrospective mouse scans (Fig. 3). Our results show radiomics features extracted from µCT scans have higher ICC scores compared to CBCT (Fig. 4 & Supplementary Table 2). For both scanners, the number of reliable features was highest for the lung (CBCT=580, µCT=734, Fig. 4A) and lowest for bone (CBCT=110, µCT=560), potentially due to increased imaging artefacts associated with higher densities. CBCT scans are more greatly affected due to their reduced image quality in comparison to µCT scanners. The NGTDM feature class has the lowest reliability across all tissues for CBCT. GLCM, GLRLM, GLSZM and GLDM features are the most reliable on both scanners for lung tissue, and first order features are the most reliable for CBCT for heart tissue.

Voxel resampling is a common normalisation step that can improve transferability of radiomics results across scanners as it is completed after scans are acquired. Voxel sizes were normalised across CBCT and µCT scanners to 0.26 mm (Fig. 4B). Similar to the results in Fig. 4A, an inverse relationship is observed between tissue density and feature reliability with ICC scores highest in the lung and lowest in the bone. For µCT images, an adjusted voxel size of 0.26 mm resulted in less reliable features across lung, heart, and bone (732, 568 and 450 features, respectively) compared to the original voxel size (734, 664, and 560 features, respectively).

### 3.3 Identification of overlapping reliable features between CBCT and µCT scanners

**Fig. 5:**
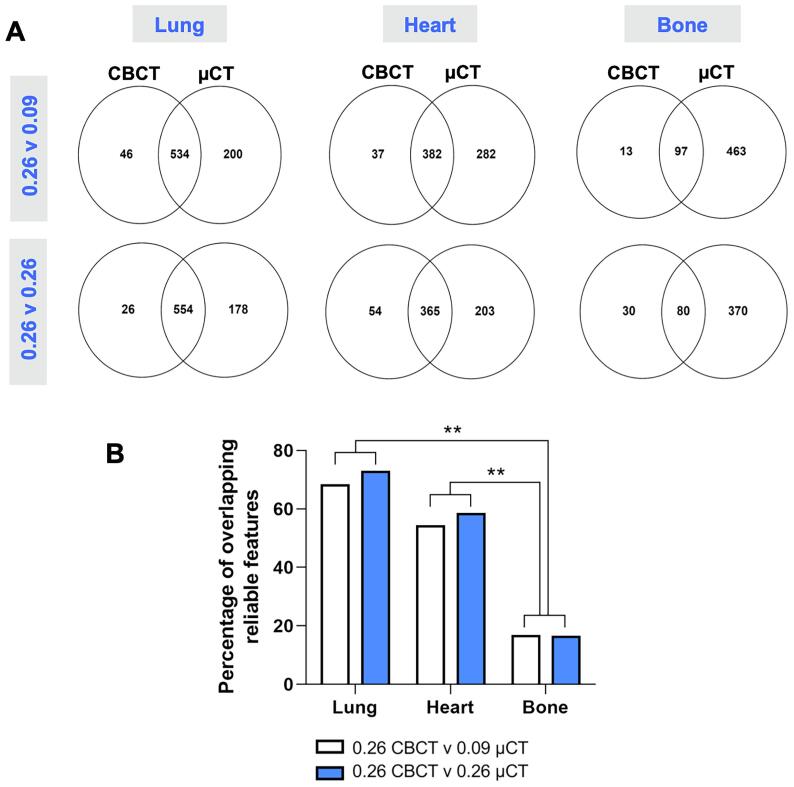
Comparative analysis of reliable radiomics features (ICC >0.8) in the lung, heart, and bone using CBCT and µCT scanners. Scan-rescan tests were conducted on both scanners and the radiomics features were extracted using slice thicknesses of 0.09 mm on µCT, and 0.26 mm on both scanners. **Panel A** shows the number of overlapping reliable features between the scanners for the different tissues and slice thicknesses. **Panel B** summarises the percentages of overlapping features for the different tissues.

Reliable features (ICC>0.8) from both scanners were compared for the lung, heart, and bone, to identify features that could be stable for comparative analysis (Fig. 5A, Supplementary File 2). This was completed for both original and normalised voxel sizes. Overall, µCT produced more reliable features than CBCT and overlapping features between both scanners decreased as tissue density increased. Normalisation improved the percentage overlap of features for the lung (73 %) and heart (59 %) (Fig. 5B). These data suggest that harmonisation of datasets may improve both the transferability and reliability of features for comparison of preclinical CBCT and µCT scanners.

### 3.4 Identification of radiomics features specific to different tissue densities

**Fig. 6:**
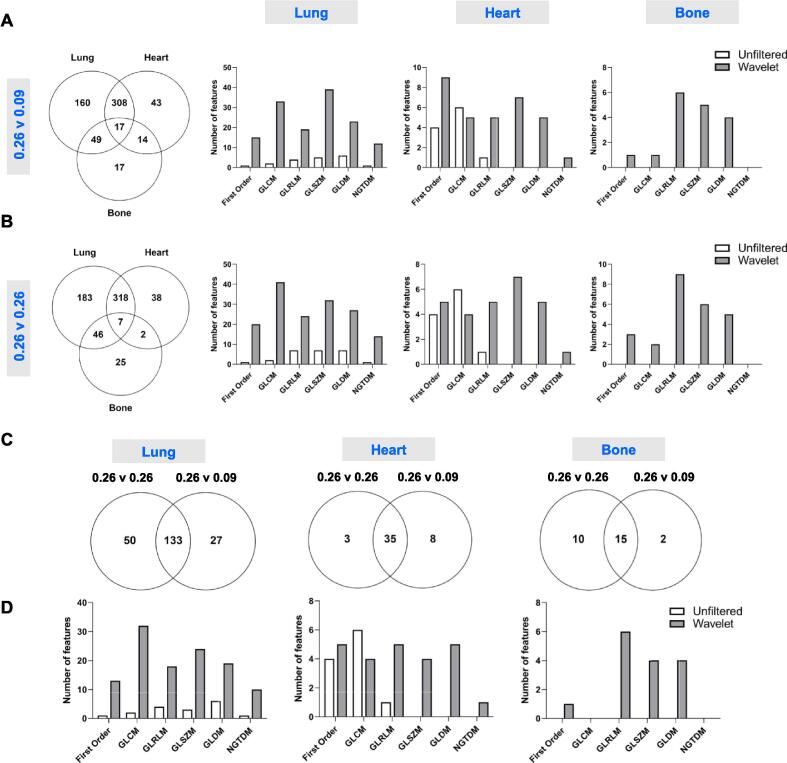
Identification of tissue-specific radiomics features for the lung, heart, and bone. Overlapping reliable radiomics features between scanners in each tissue were compared to identify tissue-specific features. Comparisons were performed at different voxel sizes of 0.26 mm and 0.09 mm **(Panel A)** or at the equivalent voxel size of 0.26 mm **(Panel B)**, and tissue-specific features were categorised by feature class for lung, heart, and bone. Shared tissue-specific features identified for each tissue at original and normalised voxel sizes were compared **(Panel C)** and the overlapping features were categorised by feature class **(Panel D)**.

Overlapping features for each tissue from Fig. 5 were compared to identify radiomics features that are reliable, transferable across both scanners, and potentially tissue density-specific (Fig. 6). These which overlapped across original and normalised methods were determined as specific to each tissue density (Fig. 6C) and radiomics signatures for the lung (133 features), heart (35 features) and bone (15 features) were determined (Supplementary File 3). These signatures may provide comparable reference features for normal or healthy tissue and alterations to these features may be used to detect damage or disease.

## 4 Discussion

Currently, preclinical radiomics studies have been performed at single institutions and with limited external validation of results. Multi-centre comparisons are required to highlight potential scanner-dependent differences and thus improve the knowledge transfer within the radiomics community [29], [30]. This study is the first to compare radiomics outputs across two CT-based preclinical imaging modalities. The reliability of radiomics features was assessed with the rationale that features with low reliability are not stable and may subsequently lead to poor predictive models [31]. Normalisation was trialled through alteration of voxel size in pre-processing steps and comparable tissue-density specific features were identified for the lung, heart, and bone. The presented results show promise for the transferability of radiomics data across CT-based scanners yet emphasise the need for thorough reliability comparisons.

CBCT and CT scanners are both instrumental within oncology for patient diagnosis, treatment planning (CT), and pre-treatment positioning (CBCT) [32]. It is pertinent that both CT-based scanners can be used, and results interchanged for radiomics analysis [33], [34], [35]. To-date only a handful of clinical studies have directly compared radiomics outputs acquired on CBCT and CT scanners [36], [37], [38]. These studies have demonstrated the feasibility of correlating CBCT and CT radiomics outputs, yet further work is required to develop prognostic imaging biomarkers. In this study we have used their preclinical counterparts, typically used for translational research [13], [39], to assess the feasibility of comparing features across imaging modalities. Our results support the hypothesis that some radiomics features are reliable and interchangeable between CT-based scanners.

Reliability and robustness tests typically use “coffee break” style scan-rescan analysis, two scans acquired on the same scanner after a short time period. Results from scan-rescan analysis are not generalizable and need to be conducted for individual scanners [40], [41], [42]. In this study, scan-rescan analysis was used to identify differences between scans on each scanner at different imaging energies and identify and remove unreliable radiomics features [19]. As CBCT scans have reduced image quality [32], [43], there is an increased level of scattering, beam hardening artifacts, and reduced accuracy of Hounsfield units (HU) which may have implications on the extraction of stable radiomics features at lower energies. However, some features are robust to this noise when imaging and analysis protocols are standardised [5], [44], [45], [46]. We have shown here and from previous work that preclinical radiomics is suspectable to changes in imaging energies and should be standardised whenever possible [19]. From phantom analysis there was more reliable features extracted from CBCT scans than µCT. This may be due to the lower quality of CBCT and when analysing a uniform phantom, but importantly this does not hold true for *in vivo* tissue analysis which have increased heterogeneity.

Segmentation, and in particular differences in segmentation volumes, is one of the main sources of variability within the radiomics workflow. This is particularly evident within oncology with the irregularity in tumour sizes. As a result, features correlated with volume could be falsely detected as predictive. Features that have been shown to correlate or heavily depend on volume may not provide any addition information and could be removed during feature reduction methods [5], [8], [19], [47]. Some radiomics features (54) have been shown to directly correlate with increasing volume on preclinical CBCT scans [19], this has yet to be validated for µCT scanners. Our results show that using a range of small segmentation volumes (44 – 238 mm^3^) does not significantly influence the reliability of radiomics features and first order & GLCM feature types may be most resistant to changes in segmentation volume (Fig. 2B).

Harmonisation methods can overcome variabilities by transforming imaging data [7], [6]. CT scans can be harmonised through the adjustment of HU values to standardise tissue contrast, however this was not feasible in our study as CBCT data from the SARRP are given in proprietary CT numbers and the accurate conversion to mass density is not routinely performed [48]. However, as radiomics textural outputs are voxel size and gray-level discretisation dependent, voxel resampling may reduce variabilities across the two systems [11], [41], [49]. Our results showed that transforming data through the harmonisation of slice thicknesses was beneficial for comparison of lower-density tissues (lung). Yet, there were marginally fewer comparable reliable features for higher-density tissues (heart and bone). These data could be due to additional artefacts or increased homogeneity in higher density tissue that could reduce the subtle variability in the radiomics features. Some radiomics features are more dependent on voxel size in comparison to gray-level discretisation and studies should take into consideration tissue density during normalisation steps [11].

The use of preclinical models to develop radiomics signatures is still a relatively new concept and requires continual optimisation. Preclinical models are pivotal within oncology research to recapitulate the underlying biology of human disease, yet mice are not mini humans [50], [51]. A recent study has shown strong correlation between radiomics features extracted from mouse and patient datasets [52], supporting the use of preclinical models to develop radiomics signatures in controlled and standardised settings. Our study has identified 133, 35 and 15 reliable features associated with the lung, heart and bone tissues in mice across two CT-based scanners. These signatures could be clinically validated to feed into pipelines applying radiomics biomarkers to differentiate between normal and abnormal tissues (e.g. tumours).

Despite compelling evidence that many reliable radiomics features can be compared across the two preclinical CT-based scanners, our study had a few limitations. These included the lack of movement of the phantom between scan and rescan and the imaging of the phantom on the same day. However, mouse scans were acquired over several days which would account for any movement and day-to-day scanner variation. Phantom analysis showed that CBCT images had more reliable radiomics features compared to µCT scans. These data are unexpected due to the differences in quality between these scanners yet may be due to the higher level of uniformity in the phantom materials than tissues *in vivo*. Some radiomics studies have increased the complexity of phantom analysis by using textural phantoms like the Credence Cartridge Radiomics (CCR) phantom [29]. The CCR phantom represents tissues which are minimally to highly varied in texture and has been used to provide insight on the impact of scanner protocols on radiomics features [29], [46], [53]. Another limitation of this study was different cohorts of mice were imaged on each scanner. To minimise uncertainties, genetically comparable immunodeficient mouse strains were used, which importantly reflects heterogeneity in patient populations. Additionally, movement on scans from motion artefacts could potentially affect analysis however, this is likely to be minimal given the small range of motion even for mobile organs (e.g. lung) that is estimated to be <5 mm [54], [55]. These limitations could be addressed by using 4-D CT scanners or gated image acquisition: however, both approaches are not routinely available for preclinical CT imaging systems.

We demonstrated variations in the reliability of radiomics features across two CT-based preclinical scanners. Our results emphasise that to improve the predictive potential of radiomics features it is paramount to use reliable radiomics features across scanners. Despite significant differences between the scanners and imaging parameters e.g. imaging protocols, geometry and intensity range, normalisation steps can be useful to improve the comparability of CBCT and µCT scans e.g. standardisation of imaging energy and pre-processing factors (voxel size). Our results have identified tissue density-specific radiomics signatures that are transferable and reliable for the lung, heart, and bone. Suggesting that preclinical CBCT and µCT scanners can both be used, and results interchanged for the investigation and development of radiomics imaging biomarkers. Our data indicate the potential need to consider optimising CT imaging protocols not only for physical parameters (contrast and noise) but for downstream quantitative radiomics analysis.

## Funding

KHB is supported by a Training Fellowship from the National Centre for the Replacement Reduction and Refinement of Animal in Research (NC3Rs, NC/V002295/1). BNK, CC, AB, KTB are funded by the Higher Education Authority North-South Research Programme 2021 RadCOL. ATB would like to acknowledge previous support from Science Foundation Ireland (SFI) and the European Regional Development Fund (ERDF) (18/RI/5759 & 13/RC/2073).

## Declaration of Competing Interests

The authors declare that they have no known competing financial interests or personal relationships that could have appeared to influence the work reported in this paper.

## Figures and Tables

**Supplementary Fig. 1:**