# Supplementary material for: A comparative analysis of preclinical computed tomography radiomics using cone-beam and micro-computed tomography scanners
Source: Phys Imaging Radiat Oncol. 2024 Jul 23;31:100615. doi: 10.1016/j.phro.2024.100615 (PMC11328005; doi:10.1016/j.phro.2024.100615)
Supplement: Supplementary Data 4 [file mmc4.docx]

|  | | **QUB** | **RCSI** |
| --- | --- | --- | --- |
| **Image Modality** | | CBCT | µCT |
| **Manufacturer** | | Xstrahl Life Sciences | Revvity (Perkin Elmer) |
| **Platform** | | SARRP | Quantum GX2 |
| **Device** | | Tesla C2075 | Rigaku |
| **Detector Panel** | | Perkin Elmer XRD 0822 AP3 | Perkin Elmer Flat Panel CMOS |
| **Imaging Energy (kVp)** | | 40, 60 | 40, 60 |
| **Current (mA)** | | 0.8 | 0.088 |
| **Imaging time (seconds)** | | 60 | 240 |
| **Current exposure time (mAs)** | | 48 | 21.12 |
| **Projections** | | 0° - 359° | 0° - 359° |
| **Slice Thickness (mm)** | | 0.26 | 0.09 |
| **Pixel Spacing (mm)** | | 0.26 | 0.09 |
| **Reconstruction** | | Backprojection with filtering | Backprojection with filtering |
| **Filtering** | | Hamming | Hamming |
| **Intensity Range** | | 0 – 32,770 | -1,000 – 1,500 HU |
|  | **Lung** | 10,000 – 11,000 | -400 – -250 |
|  | **Heart** | 12,000 – 15,000 | 0 – 500 |
|  | **Bone** | 15,000 – 32,000 | 500 – 1,500 |

**Supplementary Table 1:** A summary of CBCT and µCT scanner details and experimental parameters for comparative analysis.
