# Supplementary material for: A comparative analysis of preclinical computed tomography radiomics using cone-beam and micro-computed tomography scanners
Source: Phys Imaging Radiat Oncol. 2024 Jul 23;31:100615. doi: 10.1016/j.phro.2024.100615 (PMC11328005; doi:10.1016/j.phro.2024.100615)
Supplement: Supplementary Data 5 [file mmc5.docx]

|  | | | **ICC value**  **> 0.8** | **Reliability** | **ICC intervals** | **Number of features** |
| --- | --- | --- | --- | --- | --- | --- |
| **CBCT** | **Lung** | Unfiltered features (original) | **48** | Excellent | > 0.9 | 32 |
|  |  |  |  | Good | 0.8 – 0.9 | 16 |
|  |  | Filtered features  (wavelet) | **532** | Excellent | > 0.9 | 385 |
|  |  |  |  | Good | 0.8 – 0.9 | 147 |
|  | **Heart** | Unfiltered features (original) | **33** | Excellent | > 0.9 | 24 |
|  |  |  |  | Good | 0.8 – 0.9 | 9 |
|  |  | Filtered features  (wavelet) | **386** | Excellent | > 0.9 | 297 |
|  |  |  |  | Good | 0.8 – 0.9 | 89 |
|  | **Bone** | Unfiltered features (original) | **0** | Excellent | > 0.9 | 0 |
|  |  |  |  | Good | 0.8 – 0.9 | 0 |
|  |  | Filtered features  (wavelet) | **110** | Excellent | > 0.9 | 79 |
|  |  |  |  | Good | 0.8 – 0.9 | 31 |
| **µCT** | **Lung** | Unfiltered features (original) | **61** | Excellent | > 0.9 | 50 |
|  |  |  |  | Good | 0.8 – 0.9 | 11 |
|  |  | Filtered features  (wavelet) | **673** | Excellent | > 0.9 | 649 |
|  |  |  |  | Good | 0.8 – 0.9 | 24 |
|  | **Heart** | Unfiltered features (original) | **62** | Excellent | > 0.9 | 49 |
|  |  |  |  | Good | 0.8 – 0.9 | 13 |
|  |  | Filtered features  (wavelet) | **602** | Excellent | > 0.9 | 551 |
|  |  |  |  | Good | 0.8 – 0.9 | 51 |
|  | **Bone** | Unfiltered features (original) | **17** | Excellent | > 0.9 | 15 |
|  |  |  |  | Good | 0.8 – 0.9 | 2 |
|  |  | Filtered features  (wavelet) | **543** | Excellent | > 0.9 | 510 |
|  |  |  |  | Good | 0.8 – 0.9 | 33 |

**Supplementary Table 2:** A summary of the reliable features extracted from CBCT and µCT preclinical scanners across different tissue densities. Radiomics analysis was performed at the original slice thicknesses of 0.26 mm and 0.09 mm for CBCT and µCT, respectively, for the lung, heart, and bone. Reliable features (ICC > 0.8) were identified and categorised into unfiltered and filtered (wavelet) for each tissue.
